# Genome-Wide Analysis of the Banana NBS Gene Family and Expression Profiling of the Fusarium Wilt Resistance Gene *MamRGA2* in Response to Defense-Related Phytohormones

**DOI:** 10.3390/genes17060700

**Published:** 2026-06-16

**Authors:** Ana N. Roblero-Aguilar, Gabriel Lizama-Uc, Carlos Alberto Puch-Hau, Virginia Aurora Herrera-Valencia, Sergio García-Laynes, Jorge A. Tzec-Interián, Marta G. Lizama-Gasca, Ileana Cecilia Borges-Argaez, Santy Peraza-Echeverria

**Affiliations:** 1 Unidad de Biotecnología, Centro de Investigación Científica de Yucatán, Calle 43 No. 130 x 32 y 34, Colonia Chuburná de Hidalgo, Mérida C.P. 97205, Yucatán, Mexico; ananury.ra@gmail.com (A.N.R.-A.); vicky@cicy.mx (V.A.H.-V.); sergio.laynes@cicy.mx (S.G.-L.); jorgetzec@gmail.com (J.A.T.-I.); mar_10914@hotmail.com (M.G.L.-G.); cecilia@cicy.mx (I.C.B.-A.); 2 Departamento de Ingeniería Química-Bioquímica, Tecnológico Nacional de México Campus Instituto Tecnológico de Mérida, Avenida Tecnológico S/N Km. 4.5, Mérida C.P. 97118, Yucatán, Mexico; gabriel.lu@merida.tecnm.mx; 3 Tecnológico Nacional de México Campus Instituto Tecnológico Superior de Valladolid, Carretera Valladolid-Tizimín Km. 3.5 Tablaje Catastral No. 8850, Valladolid C.P. 97780, Yucatán, Mexico; carlos.ph@valladolid.tecnm.mx

**Keywords:** banana, NBS genes, *MamRGA2*, Foc TR4, jasmonate

## Abstract

**Background/Objectives:** Banana (*Musa* spp.) production is severely threatened by Fusarium wilt caused by *Fusarium oxysporum* f. sp. *cubense* tropical race 4 (Foc TR4), highlighting the need to identify genetic determinants of resistance. **Methods:** We performed a genome-wide analysis of NBS genes in *Musa acuminata* ssp. *malaccensis*, including phylogenetic, chromosomal, and microsynteny analyses. The genomic context and promoter regions of *MamRGA2* were characterized, its response to defense-related phytohormones was evaluated by RT-qPCR, and its protein structure was predicted by homology modeling. **Results:** A total of 118 NBS genes were identified. Notably, we report for the first time in banana two NBS genes encoding proteins with integrated domains, corresponding to an ATP-binding cassette (ABC) transporter and a Nuclear Factor Y subunit A (NF-YA) transcription factor. Chromosomal mapping revealed a marked enrichment of NBS genes on chromosome 3, which harbors *MamRGA2*, an NBS gene associated with resistance to Foc TR4. RT-qPCR analyses showed that *MamRGA2* is strongly induced by exogenous methyl jasmonate (MeJA) in the resistant wild genotype but not in a susceptible Cavendish cultivar, a pattern associated with divergence in promoter sequences between the two genotypes. Structural modeling suggested that the MamRGA2 protein possesses features consistent with a resistosome-like architecture. **Conclusions:** Overall, these findings expand current knowledge of NBS gene diversity in banana and provide a framework for future studies aimed at elucidating the molecular mechanisms underlying resistance to Foc TR4.

## 1. Introduction

Banana (*Musa* spp.) is one of the most important food crops worldwide, with an estimated annual production of approximately 183 million tons [1], playing a crucial role in food security and in the economies of many tropical and subtropical countries [2]. However, banana production is severely threatened by numerous phytopathogens, among which the soil-borne fungus *Foc* TR4, the causal agent of Fusarium wilt, stands out as one of the most devastating [3,4]. This pathogen infects the roots of susceptible cultivars, including Cavendish, causing vascular wilt that ultimately leads to plant death [5]. The persistence of *Foc* TR4 chlamydospores in soil for decades, combined with the ineffectiveness of chemical control strategies, underscores the urgent need for alternative approaches [6,7]. The deployment of resistance (R) genes represents one of the most effective and sustainable solutions, as R proteins recognize pathogen invasion and activate innate immune responses, providing durable protection without the environmental costs associated with agrochemicals [8].

Plant immunity consists of two major layers: pattern-triggered immunity (PTI), mediated by cell surface receptors such as receptor-like kinases (RLKs) and receptor-like proteins (RLPs), and effector-triggered immunity (ETI), which is mediated by intracellular R proteins, predominantly nucleotide-binding leucine-rich repeat (NLR) receptors [9,10]. NLR proteins represent the largest class of plant R proteins and are essential for resistance against a broad spectrum of pathogens. To date, more than 200 functional NLR genes have been cloned from diverse plant species, conferring resistance to viruses, bacteria, fungi, nematodes, parasitic plants, and insects [11,12]. NLRs detect pathogen effectors either directly or indirectly and trigger rapid and robust defense responses [13], including the hypersensitive response (HR), a localized programmed cell death that restricts pathogen spread. HR can also activate salicylic acid (SA)-dependent systemic acquired resistance (SAR), which provides long-lasting protection against subsequent infections. Jasmonic acid (JA) represents another key phytohormone involved in NLR-mediated defense signaling, particularly in induced systemic resistance (ISR) and responses to necrotrophic pathogens and herbivores [14].

Structurally, NLR proteins possess a modular architecture consisting of a central NBS domain and a C-terminal LRR domain. The NBS domain mediates signal activation and is the most conserved region, whereas the LRR domain is highly variable and primarily responsible for effector recognition [15]. Based on their N-terminal domains, NLRs are classified into coiled-coil NLRs (CNLs), RPW8-type NLRs (RNLs), and Toll/interleukin-1 (TIR) receptor NLRs (TNLs) [16,17,18]. Many NLRs also harbor non-canonical integrated domains (IDs), which are thought to function as molecular decoys that mimic host targets of pathogen effectors, thereby enabling indirect effector recognition [19,20]. The potential of IDs for crop improvement has been demonstrated by Cesari et al. [21], who showed that replacing the ID of the rice NLR RGA5 with a nanobody expanded its recognition spectrum, providing proof of concept for the engineering of novel immune specificities.

Numerous wild species and subspecies of the genus *Musa* produce fruits that are unsuitable for human consumption due to low pulp content, abundant seeds, and poor palatability—traits linked to their non-domesticated state. Nevertheless, many wild genotypes display strong resistance to major diseases, including Foc TR4, highlighting their value as genetic resources for banana improvement [22,23]. Over the past 15 years, several wild *Musa* genomes have been sequenced, enabling genome-wide analyses of gene families involved in plant immunity [24] and accelerating the identification of candidate resistance genes. The genome of the wild subspecies *M. acuminata* ssp. *malaccensis* [25] was the first to be sequenced and remains one of the most extensively studied banana genomes [26]. Genome-wide analyses of the NBS gene family in version 4 of this genome have reported between 97 and 116 members [27,28]; however, these studies did not investigate whether banana NBS proteins harbor integrated domains (IDs), which are increasingly recognized as important evolutionary features that can expand pathogen recognition capabilities in plant NLRs. Furthermore, despite the importance of *MamRGA2* as the only cloned banana resistance gene shown to confer field resistance to Foc TR4 [29], its genomic context within the NBS repertoire, regulatory characteristics, and structural features have not been comprehensively investigated.

To expand current knowledge of NBS gene structure in banana and to investigate *MamRGA2* within a genomic context that remains poorly understood, we performed a comprehensive genome-wide analysis of the NBS gene family in version 4 of the *M. acuminata* ssp. *malaccensis* genome sequence. This study included structural characterization of NBS genes, chromosomal distribution, prediction of subcellular localization, and phylogenetic relationships with known R genes. In addition, we analyzed the genomic organization of *MamRGA2*, its transcriptional response to the defense-related phytohormones SA and JA, and cis-regulatory elements within its promoter region. Finally, we generated a structural model of the *MamRGA2* resistosome. The findings of this study expand the repertoire of NBS genes in banana and provide new insights into the molecular and evolutionary basis of resistance in *M. acuminata* ssp. *malaccensis*. Moreover, they offer valuable resources for developing biotechnological strategies aimed at generating banana cultivars resistant to Foc TR4 and other major phytopathogens.

## 2. Materials and Methods

### 2.1. Biological Material and Phytohormone Treatments

Ex vitro plantlets of *M. acuminata* ssp. *malaccensis* (ITC1060) and *M. acuminata* cv. Grand Nain (group AAA, subgroup Cavendish) were cultivated for 31 days in individual pots containing a sterile mixture of peat moss and vermiculite (1:1, *v/v*). Plants were maintained under controlled environmental conditions with a 12 h light/12 h dark photoperiod, a light intensity of 100 μmol m^−2^ s^−1^, a temperature of 25 ± 1 °C, and a relative humidity of approximately 70%. Phytohormone treatments were arranged in a completely randomized design, with plants randomly assigned to treatment and mock-control groups. Phytohormone treatments were conducted as two independent assays for each genotype: salicylic acid (SA) and methyl jasmonate (MeJA). For each assay, both the phytohormone-treated and mock-control groups consisted of three independent biological replicates, resulting in a total of 12 independent plants per genotype. Plants were sprayed on the abaxial surface of the first fully expanded leaf adjacent to the cigar leaf with either 5 mM salicylic acid (SA) or 100 µM methyl jasmonate (MeJA) until runoff using a handheld sprayer (Nalgene Nunc International, Rochester, NY, USA; Cat. No. 2430-0200), following the protocols described by Tzean et al. [30] and Zhao et al. [31], respectively. Based on these studies, leaf samples were collected 6 h after treatment, a time point at which marker genes associated with the SA and MeJA signaling pathways in banana are known to be induced. Each phytohormone assay was independently repeated once. Harvested tissues were immediately frozen in liquid nitrogen and stored at −80 °C until further processing.

### 2.2. Annotation of Banana NBS Genes

Genome sequences, coding sequences (CDSs), and predicted proteome of *M. acuminata* ssp. *malaccensis* (version 4) were downloaded from the Banana Genome Hub repository (https://banana-genome-hub.southgreen.fr/). The corresponding sequence datasets for *M. acuminata* cv. Baxijiao (AAA group, Cavendish subgroup) [22] were obtained from the same database. Identification of genes encoding NBS domain-containing proteins in *M. acuminata* ssp. *malaccensis* was performed using a Hidden Markov Model (HMM)-based approach with HMMsearch from the HMMER v3.3.2 package [32] (http://hmmer.org/) under default parameters and an E-value cutoff of ≤0.01. The conserved NB-ARC domain profile (Pfam ID: PF00931) was used as the reference model and retrieved from the Pfam database (http://pfam.xfam.org/; accessed 27 September 2022). Candidate protein sequences were subsequently validated for the presence of the NBS domain using the combined results of SMART [33] (http://smart.embl-heidelberg.de/, accessed on 27 September 2022) and InterProScan v5.54-87 [34] (https://www.ebi.ac.uk/interpro/search/sequence/, accessed on 27 September 2022). The presence of the coiled-coil (CC) domain was assessed using PCOILS implemented in the MPI Bioinformatics Toolkit, applying a minimum probability threshold of 0.5 and a sliding window of 28 residues (https://toolkit.tuebingen.mpg.de/tools/pcoils; accessed 17 February 2022). Finally, the theoretical isoelectric point and molecular weight of the predicted proteins were calculated using the Compute pI/Mw tool [35] (https://web.expasy.org/compute_pi; accessed on 17 February 2022).

### 2.3. Gene Structure Analysis and Identification of Conserved Protein Motifs

The exon–intron organization of NBS genes was determined using structural annotation files (GFF3) corresponding to the *M. acuminata* ssp. *malaccensis* genome. Conserved protein motifs within NBS proteins were identified using MEME v5.4.1 [36] (https://meme-suite.org/meme/tools/meme, accessed on 17 February 2022) using default parameters with a maximum of 10 motifs allowed. Integrated visualization of gene structures, and the distribution of conserved motifs was performed using the Gene Structure View module of TBtools II [37] (https://github.com/CJ-Chen/TBtools/releases, accessed on 17 February 2022), enabling comparative representation of exon–intron architecture alongside the motif composition of the encoded proteins.

### 2.4. Sequence Alignment and Phylogenetic Analysis

NBS protein sequences from *M. acuminata* ssp. *malaccensis* were aligned using the MUSCLE algorithm [38]. For phylogenetic reconstruction, the conserved NBS domain was specifically analyzed, as it is widely used in evolutionary studies of this gene family [39], due to its high structural and functional conservation. Phylogenetic analysis was performed using MEGA v11.0.13 [40] with the Neighbor-Joining (NJ) method. The Poisson substitution model was applied, and branch support was assessed using bootstrap analysis with 1000 replicates. Pairwise deletion was employed to handle sites with missing data. To provide evolutionary and functional context, 70 functionally characterized plant resistance genes were included [10] (Supplementary Table S2).

### 2.5. Subcellular Localization Prediction

The subcellular localization of the 118 NBS domain-containing proteins identified in *M. acuminata* ssp. *malaccensis* was predicted using the DeepLoc-2.1 web server [41] (https://services.healthtech.dtu.dk/services/DeepLoc-2.1/, accessed on 15 June 2024). Predictions were performed using the high-quality model with default parameters. For each protein, the predicted subcellular compartment was assigned according to the highest probability provided by the model. The resulting predictions were compiled and visualized as a heatmap using TBtools II [37], allowing comparative representation of the subcellular distribution patterns across the NBS protein family.

### 2.6. Chromosomal Distribution of NBS Genes

NBS domain-containing genes identified in *M. acuminata* ssp. *malaccensis* were mapped onto the 11 chromosomes of the reference genome according to their annotated genomic coordinates. Chromosomal distribution was visualized using MapGene2Chromosome (MG2C) v2 [42] (http://mg2c.iask.in/mg2c_v2.0/, accessed on 10 March 2024)**.** To identify gene clusters, adjacent genes located on the same chromosome and separated by an intergenic distance ≤200 kb were defined as clustered, following the criterion proposed by Jupe et al. [43].

### 2.7. Annotation of Genes Flanking MamRGA2 and Microsynteny Analysis

To identify syntenic relationships between *M. acuminata* ssp. *malaccensis* and *M. acuminata* cv. Baxijiao pairwise protein sequence comparisons were performed for chromosome 3 using LAST v1607. Microsynteny analyses were conducted in a Python v3.12.8 environment using MCScan implemented in the JCVI toolkit v1.4.24, with default parameters (c-score ≥ 0.70) [44]. Anchors were filtered to retain high-confidence gene pairs, and collinear blocks were identified using increased iterations (--iter = 3) to reflect the expected 1:3 ploidy relationship between *M. acuminata* ssp. *malaccensis* and *M. acuminata* cv. Baxijiao. Microsynteny visualization was performed using the toolkit’s built-in graphical functions, focusing on a ±200 kb genomic window flanking the *MamRGA2* locus (Macma4_03_g09360.1). Genes located within this interval were examined to infer their orthologous relationships and functional annotation. For *MamRGA2* orthologs, protein sequences were aligned with MUSCLE v3.8.425 [38], and conserved domains were predicted using InterProScan v5.74-105 [34].

### 2.8. Gene Expression Analysis by RT-qPCR

Total RNA was extracted from plants treated with SA or MeJA (see Section 2.1) using the Norgen Plant/Fungi Total RNA Purification Kit (Norgen, Thorold, ON, Canada) following the manufacturer’s instructions. Complementary DNA (cDNA) was synthesized by reverse transcription using SuperScript III reverse transcriptase (Thermo Fisher Scientific, Waltham, MA, USA) according to the supplier’s protocol. Gene-specific primers for *MamRGA2*, *MaDLO1*, *MaLOX1*, and the banana ribosomal *25S* rRNA reference gene were used for quantitative analysis (Supplementary Table S5). *MaDLO1* was used as a marker of the SA signaling pathway [30] whereas *MaLOX1* served as a marker of JA signaling pathway [31]. The banana ribosomal *25S* rRNA gene was used as an internal reference for normalization [45]. RT-qPCR reactions were performed on a StepOnePlus™ Real-Time PCR System (Thermo Fisher Scientific) using SYBR Green PCR Master Mix (Thermo Fisher Scientific). Relative transcript levels were calculated using the 2^−ΔΔCt^ method [46]. Three independent biological replicates, each with three technical replicates, were used for statistical analysis. Data normality was assessed using the Anderson–Darling test. Differences between treatments were evaluated using a two-tailed Welch’s *t*-test. All statistical analyses were performed in R software v4.3.1 [47]. Differences were considered statistically significant when *p* < 0.05. The RT-qPCR experiment was independently repeated once.

### 2.9. cis-Regulatory Element Analysis and Three-Dimensional Modeling of the MamRGA2 Protein

Promoter regions of the *MamRGA2* gene (Macma4_03_g09360.1) from *M. acuminata* ssp. *malaccensis* and its ortholog *MacRGA2* (BXH1_00016606-R1) from *M. acuminata* cv. Baxijiao were analyzed to identify *cis*-regulatory elements (CREs). A 2000 bp sequence upstream of the translation start site, was retrieved from the Banana Genome Hub (https://banana-genome-hub.southgreen.fr/). CRE identification and annotation were performed using the PlantCARE database (https://bioinformatics.psb.ugent.be/webtools/plantcare/html/; accessed on 15 June 2024).

The three-dimensional structure of the MamRGA2 protein was predicted by homology modeling using the SWISS-MODEL server (https://swissmodel.expasy.org/). To approximate the activated state of the receptor, the pentameric resistosome structure of the CNL protein ZAR1 from *Arabidopsis thaliana* (PDB ID: 6J5T; resolved at 3.4 Å by cryo-EM) was used as the structural template. ZAR1 was selected as the closest available structural reference because MamRGA2 is also predicted to encode a CNL protein. Model quality was assessed using the GMQE score (0.27). The selected template shared 28.81% sequence identity with MamRGA2.

## 3. Results

### 3.1. Identification of Banana NBS Genes

Genome-wide analysis of the *M. acuminata* ssp. *malaccensis* genome identified 118 genes encoding NBS domain-containing proteins, representing approximately 0.32% of all predicted genes (Table 1 and Supplementary Table S1). Subfamily classification revealed that 79 proteins (66.94%) possess the canonical CC-NBS-LRR architecture, including MamRGA2 (Macma4_03_g09360.1), which is associated with resistance to Foc TR4. This finding establishes CC-NBS-LRR proteins as the predominant subfamily (Table 1). Importantly, this study reports for the first time the presence of two IDs in NBS proteins from *M. acuminata* ssp. *malaccensis*. The first, corresponding to an ABC transporter domain, was located at the N-terminus of the protein encoded by Macma4_07_g08620.1, whereas the second, corresponding to an NF-YA transcription factor domain, was identified at the N-terminus of the protein encoded by Macma4_08_g32130.1. Both proteins belong to the ID-CC-NBS-LRR subfamily (Table 1). Additionally, a gene encoding an RPW8-NBS-LRR protein was identified, a domain organization not reported in previous banana studies [27,28].

### 3.2. Structural and Phylogenetic Analysis of Banana NBS Genes

**Figure 1:**
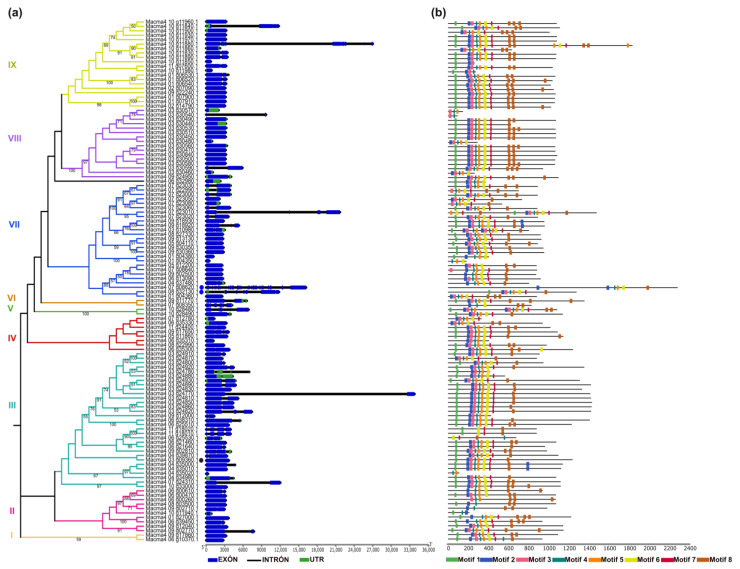
Gene structure and phylogenetic relationships of the NBS gene family in Musa *acuminata* ssp. *malaccensis*. (**a**) Structural organization of the 118 NBS-LRR genes, showing exons (blue boxes), introns (black lines), and untranslated regions (UTRs; green boxes). A phylogenetic tree was constructed using the Neighbor-Joining method based on the NBS domain, spanning from the P-loop to the GLPLA motif, with 1000 bootstrap replicates. Colors indicate the nine major clades identified. The *MamRGA2* gene is highlighted with a black circle, whereas proteins containing integrated domains (IDs) are indicated by blue circles. (**b**) Distribution of conserved protein motifs in the corresponding NBS proteins, identified using MEME; each color represents a distinct motif.

Gene structure analysis (Figure 1a) revealed substantial variability in exon–intron organization and untranslated regions (UTRs) among NBS genes of *M. acuminata* ssp. *malaccensis*. A total of 55 genes (46.6%) were intronless, including the resistance gene *MamRGA2*, whereas 42 genes (35.6%) contained a single intron. In addition, 15 genes (12.7%) harbored three or more introns. The gene Macma4_07_g08620.1 exhibited the most complex architecture, containing 25 introns. This gene corresponds to the NBS protein carrying an N-terminal ABC transporter-type integrated domain.

Analysis of conserved protein motifs (Figure 1b; Supplementary Figure S1) identified eight major motifs distributed across the encoded NBS proteins. Motif 1 is associated with the CC domain, which is involved in oligomerization and activation of immune signaling. Motifs 2–7 correspond to the NBS domain responsible for ATP binding and hydrolysis, whereas motif 8 is associated with the LRR domain implicated in effector recognition. Most NBS proteins contained combinations of these motifs: 102 proteins (86.4%) harbored LRR-related motifs, and 91 proteins (77.1%) contained CC-associated motifs.

Phylogenetic analysis of the 118 NBS proteins grouped them into nine major clades (I–IX), along with a single singleton gene (Figure 1a). Clade III was the most abundant, comprising 31 genes (26.3% of the total), including the resistance gene *MamRGA2*. The two genes harboring integrated domains, one containing an ABC transporter domain and the other an NF-YA transcription factor domain, clustered within clade VII.

**Figure 2:**
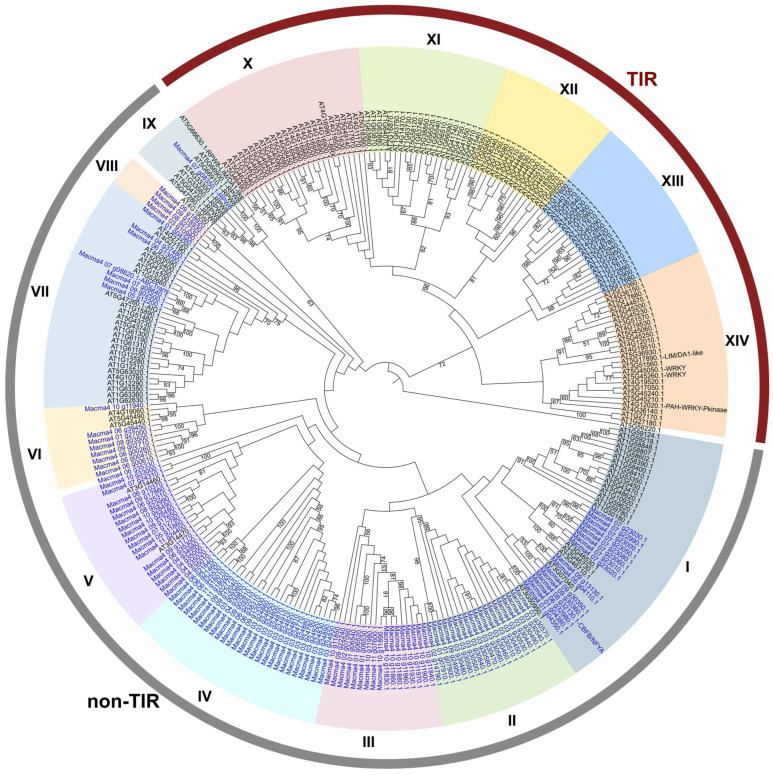
Phylogenetic tree of NBS proteins from *Musa* acuminata ssp. *malaccensis* and *Arabidopsis thaliana*. The different clades are indicated by colors. Sequence identifiers of *M. acuminata* ssp. *malaccensis* are shown in blue, whereas those of *A. thaliana* are shown in black. The phylogenetic tree was constructed using the NBS domain spanning from the P-loop motif to the GLPLA motif, based on the Neighbor-Joining method. Numerical values on the branches represent bootstrap support percentages derived from 1000 replicates.

To place banana NBS proteins within a broader evolutionary context, a phylogenetic analysis including 166 NBS proteins from the model plant *A. thaliana* was performed (Figure 2). These *A. thaliana* proteins comprise both TIR-type and non-TIR NBS proteins. Fourteen major clades were resolved, showing a clear structural separation between TIR and non-TIR lineages. All 118 banana NBS proteins clustered exclusively within clades I–IX alongside 59 non-TIR *A. thaliana* proteins, whereas clades X–XIV contained only TIR-type proteins (107 genes).

**Figure 3:**
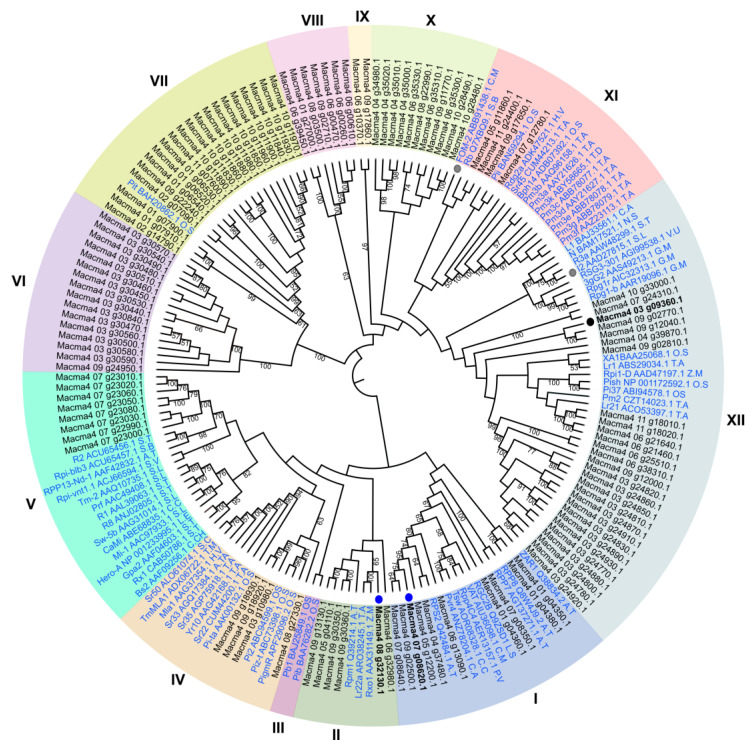
Phylogeny of NBS genes from *Musa acuminata* ssp. *malaccensis* and experimentally validated resistance (R) genes. Phylogenetic tree comprising 118 banana NBS genes and 70 functionally characterized resistance (R) genes. Genes from *M. acuminata* ssp. *malaccensis* are shown in black, whereas *R* genes are shown in blue. The *MamRGA2* gene is indicated by a black circle, while the *R* genes *Fom-2* and *I2*, associated with resistance to *Fusarium oxysporum*, are marked with gray circles; genes containing integrated domains (IDs) are highlighted with blue circles. The tree was constructed using the NBS domain region spanning from the P-loop motif to the GLPLA motif based on the Neighbor-Joining method. Numerical values on the branches represent bootstrap support percentages obtained from 1000 replicates.

To further assess evolutionary relationships with functionally validated resistance proteins, a phylogenetic tree was constructed including the 118 banana NBS proteins and 70 experimentally characterized R proteins conferring resistance to viruses, bacteria, oomycetes, fungi, nematodes, insects, or parasitic plants (Figure 3). These *R* genes were derived from 23 plant species, including seven monocots and 16 dicots (Supplementary Table S2). The analysis resolved twelve major clades, eight of which contained at least one validated *R* gene. Clades V and XII harbored the largest number of *R* genes (15 each) grouped with *M. acuminata* ssp. *malaccensis* genes, whereas clade VII contained only a single *R* gene associated with banana sequences. Notably, the gene encoding the ABC transporter-type integrated domain clustered within clade I, which included nine *R* genes, while the NF-YA-type integrated domain gene grouped within clade II, containing three *R* genes. The resistance gene *MamRGA2* was located in clade XII, showing a close phylogenetic relationship with the tomato resistance gene *I2* and proximity to clade XI, which contains the melon resistance gene *Fom-2*, both known to confer resistance to *F. oxysporum*.

### 3.3. Subcellular Localization Prediction of Banana NBS Proteins

**Figure 4:**
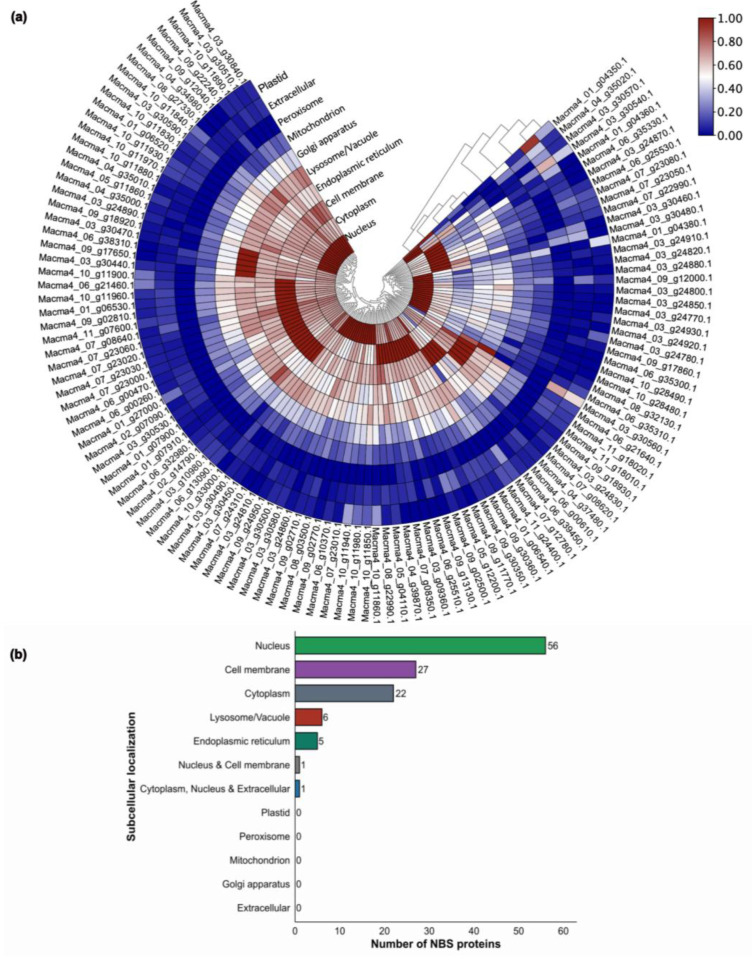
Predicted subcellular localization of NBS proteins from *Musa acuminata* ssp. *malaccensis*. (**a**) Heatmap showing the predicted subcellular localizations of the 118 NBS proteins from *M. acuminata* ssp. *malaccensis*. Red shades indicate a higher probability of localization, whereas blue shades represent a lower probability. The color scale, shown in the upper right, corresponds to predicted probability values ranging from 0 to 1. (**b**) Total number of NBS proteins predicted in each cellular compartment.

Subcellular localization prediction of the 118 NBS proteins identified in *M. acuminata* ssp. *malaccensis* (Figure 4) indicated that the nucleus is the predominant compartment, with 56 proteins assigned to this location (Figure 4b). This was followed by the plasma membrane (27 proteins), cytoplasm (22), lysosome/vacuole (six), and endoplasmic reticulum (five). Two proteins were predicted to exhibit multiple localizations: one simultaneously in the nucleus and plasma membrane, and another in the cytoplasm, nucleus, and extracellular space. No proteins were predicted to localize to the peroxisome, mitochondrion, Golgi apparatus, or exclusively to the extracellular space.

Proteins harboring IDs displayed localization patterns consistent with the functional nature of these domains. The protein containing an ABC transporter-type domain was predicted to reside in the plasma membrane, whereas the protein carrying an NF-YA transcription factor domain was localized to the nucleus. Notably, the protein encoded by *MamRGA2* was predicted to localize predominantly in the cytoplasm.

### 3.4. Chromosomal Distribution of NBS Genes in Banana

**Figure 5:**
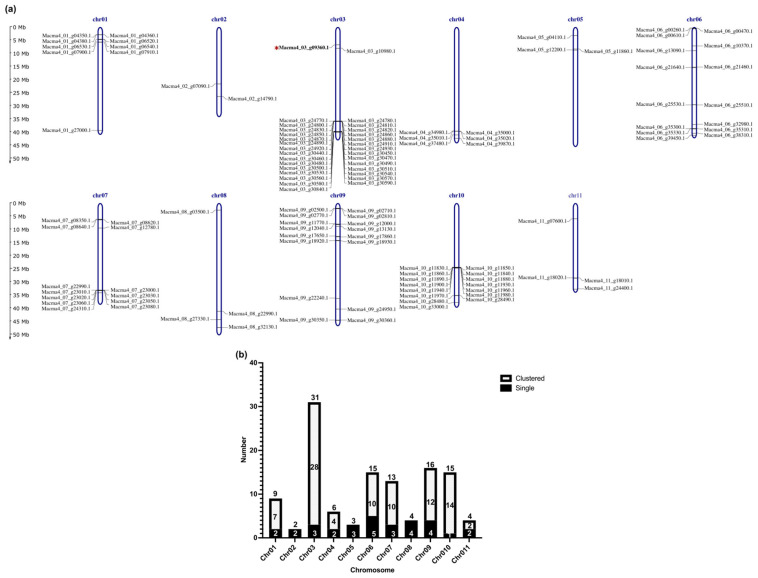
Chromosomal distribution of NBS genes identified in *Musa acuminata* ssp. *malaccensis*. (**a**) Chromosomal localization of the 118 NBS genes across the 11 chromosomes (chr01–chr11). (**b**) Total number of NBS genes per chromosome, classified according to their organization into gene clusters (textured white bars) or as singletons (black bars). An asterisk (*) indicates the location of *MamRGA2*.

The 118 NBS genes identified in *M. acuminata* ssp. *malaccensis* displayed a highly uneven distribution across the eleven chromosomes (Figure 5a and Supplementary Table S3). Chromosome 3 harbored the largest number of loci, with 31 genes (26.3% of the total), followed by chromosomes 9 (16 genes), 6 and 10 (15 genes each), 7 (13 genes), 1 (9 genes), 4 (6 genes), 8 and 11 (4 genes each), 5 (3 genes), and finally chromosome 2 (2 genes) (Figure 5b).

The resistance gene *MamRGA2*, associated with resistance to Foc TR4, was located near the upper end of chromosome 3 as a singleton. The nearest NBS gene, Macma4_03_g10980.1, lies approximately 1.6 Mb away. Comparative analysis between the CDS of the two genes revealed 46.73% identity at the nucleotide level and 25.80% at the protein level. Based on the criteria proposed by Jupe et al. [43] for defining gene clusters, 23 clusters encompassing 87 of the 118 NBS genes (74%) were identified, while the remaining 31 genes (26%) were classified as singletons (Figure 5b). Clusters were primarily located on chromosomes 3 (28 genes), 10 (14 genes), 9 (12 genes), 6 and 7 (10 genes each), 1 (7 genes), 4 (4 genes), and 11 (2 genes). No clusters were detected on chromosomes 2, 5, and 8.

### 3.5. Flanking Genes and Microsynteny Analysis of the MamRGA2 Locus Between M. acuminata ssp. malaccensis and M. acuminata cv. Baxijiao

**Figure 6:**
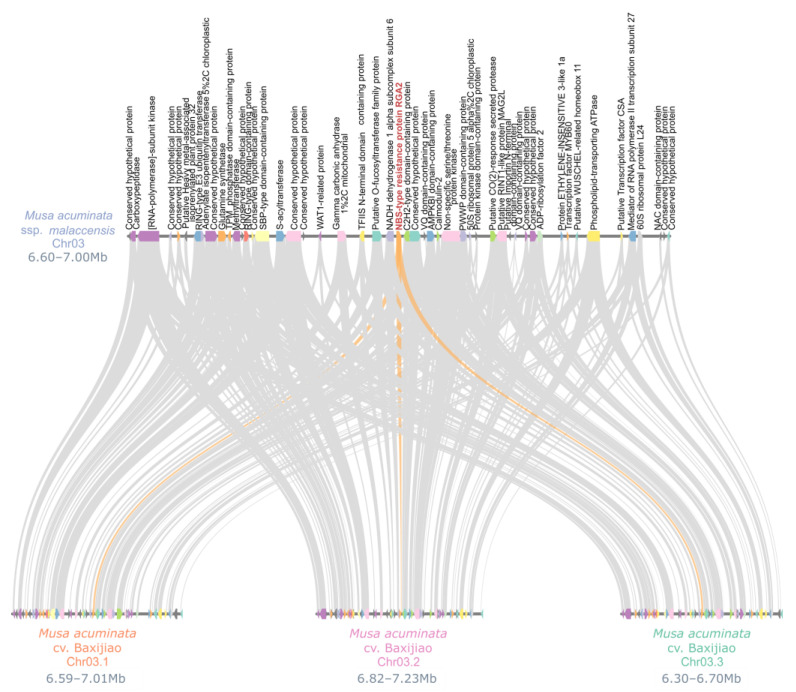
Flanking genes and microsynteny of the *MamRGA2* locus between *Musa acuminata* ssp. *malaccensis* and *Musa acuminata* cv. Baxijiao. The analysis encompassed 200 kb upstream and 200 kb downstream of the *MamRGA2* locus on chromosome 3 of *M. acuminata* ssp. *malaccensis* (Chr03: 6,600,191–7,001,525) and the corresponding syntenic regions in the Baxijiao subgenomes: Chr03.1 (6.59–7.01 Mb), Chr03.2 (6.82–7.23 Mb), and Chr03.3 (6.30–6.70 Mb). Gray lines indicate orthologous gene pairs shared between the two genotypes, whereas orange lines highlight the *MamRGA2* orthologs across the three Baxijiao subgenomes. The *MamRGA2* locus in *M. acuminata* ssp. *malaccensis* is highlighted in red.

Analysis of 200 kb upstream and 200 kb downstream of the *MamRGA2* locus on chromosome 3 of *M. acuminata* ssp. *malaccensis* (Chr03: 6,600,191–7,001,525) identified 50 genes located in the immediate genomic vicinity of *MamRGA2* (Figure 6). Of these, 22 genes (44%) were assigned to functional categories associated with biotic stress responses, including those encoding proteins such as calmodulin-2 (Macma4_03_g09410), an importin-like protein (Macma4_03_g09480), WAT1 (Macma4_03_g09310), E3 ubiquitin ligases (Macma4_03_g09180, Macma4_03_g09240), methyltransferase (Macma4_03_g09230), and transcription factors belonging to the NAC (Macma4_03_g09600), EIN3/EIL (Macma4_03_g09530), and WUSCHEL (Macma4_03_g09550) families, among others. A smaller fraction comprised genes associated with abiotic stress responses (8 genes, 16%) and other processes (7 genes, 14%). The remaining 13 genes (26%) lacked functional annotation (Supplementary Table S4).

Microsynteny analysis identified three orthologs of *MamRGA2* in *M. acuminata* cv. Baxijiao (AAA Group, Cavendish Subgroup) [22]: BXH1_00016606-R1 (Chr03.1), BXH2_00020569-R1 (Chr03.2), and BXH3_00026970-R1 (Chr03.3). The CC-NBS-LRR domain architecture was largely conserved between BXH1_00016606-R1 and BXH3_00026970-R1, whereas BXH2_00020569-R1 appeared truncated, retaining only part of the LRR domain. Among these three protein sequences, BXH1_00016606-R1 showed the highest percentage of identity relative to *MamRGA2* (97.06%).

Of the 50 flanking genes, 46 (92%) had identifiable orthologs in cv. Baxijiao, distributed across the three subgenomes Chr03.1 (6.59–7.01 Mb), Chr03.2 (6.82–7.23 Mb), and Chr03.3 (6.30–6.70 Mb). The four loci absent in cv. Baxijiao were Macma4_03_g09120, Macma4_03_g09300, Macma4_03_g09480, and Macma4_03_g09610 (Supplementary Table S4). Among these, only Macma4_03_g09480 encodes a protein with a known function, annotated as an importin-like protein.

### 3.6. Expression Analysis of MamRGA2 in Response to Defense Phytohormones SA and MeJA

**Figure 7:**
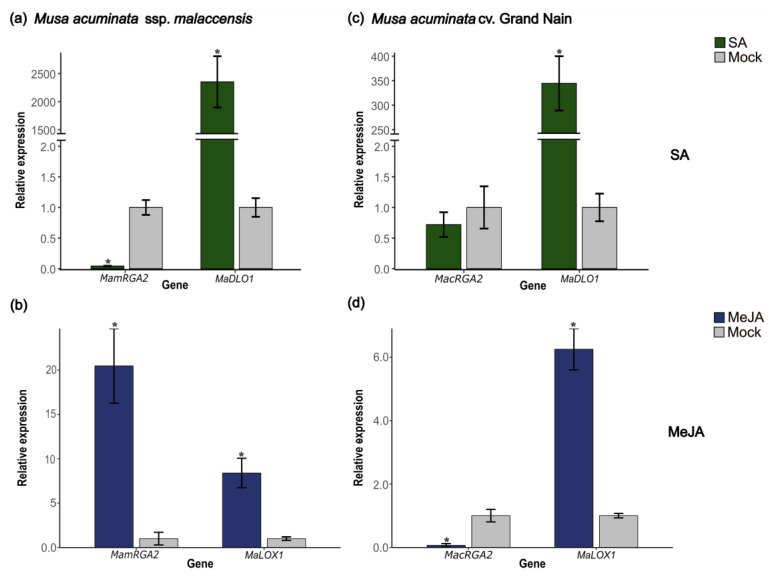
Expression profiles of *MamRGA2* and *MacRGA2* in response to the defense phytohormones salicylic acid (SA) and methyl jasmonate (MeJA) in *Musa acuminata* ssp. *malaccensis* and *M. acuminata* cv. Grand Nain, respectively. (**a**) *MamRGA2* expression in *Musa acuminata* ssp. *malaccensis* following SA treatment. (**b**) *MamRGA2* expression in *M. acuminata* ssp. *malaccensis* following MeJA treatment. (**c**) *MacRGA2* expression in cv. Grand Nain following SA treatment. (**d**) *MacRGA2* expression in cv. Grand Nain following MeJA treatment. Relative transcript levels were quantified by RT-qPCR in leaf tissues at 6 h post-treatment. The banana genes *MaDLO1* and *MaLOX1* [30,31] which are inducible by SA and MeJA, respectively, were used as markers of hormone pathway activation. Data are presented as mean ± standard deviation of three independent biological replicates. Asterisks (*) indicate statistically significant differences relative to the mock-treated control (*p* < 0.05). The experiment was independently repeated once, yielding comparable results.

Transcriptional regulation of *MamRGA2* was evaluated by RT-qPCR in the wild genotype *M. acuminata* ssp. *malaccensis* and the domesticated genotype *M. acuminata* cv. Grand Nain (AAA group, Cavendish subgroup), 6 h after treatments with the defense-related phytohormones salicylic acid (SA) and methyl jasmonate (MeJA) (Figure 7). The genes *MaDLO1* [30] and *MaLOX1* [31] were used as positive markers of SA- and MeJA-mediated signaling pathways, respectively. The *MamRGA2* ortholog in the Cavendish cultivar was named *MacRGA2*.

Analysis of basal expression under mock conditions revealed consistently higher expression of *RGA2* in *M. acuminata* ssp. *malaccensis* than in cv. Grand Nain. Specifically, *MamRGA2* transcript levels were 6.0 ± 2.94-fold higher in the mock samples from the MeJA experiment and 4.01 ± 1.53-fold higher in the corresponding mock samples from the SA experiment. In *M. acuminata* ssp. *malaccensis*, SA treatment resulted in a significant reduction of MamRGA2 expression relative to the control, whereas MeJA treatment induced a significant upregulation, reaching a 20.47 ± 4.2-fold increase. Treatment efficacy was confirmed by the marker genes: *MaDLO1* increased 2352 ± 456-fold after SA application, and *MaLOX1* showed an 8.38 ± 1.66-fold increase in response to MeJA, indicating activation of the respective signaling pathways (Figure 7a,b). In contrast, in cv. Grand Nain, *MacRGA2* did not exhibit increased expression under either hormone treatment. Nevertheless, the marker genes responded strongly, with *MaDLO1* and *MaLOX1* increasing 344 ± 55.38-fold and 6.25 ± 0.65-fold, respectively, confirming activation of SA and MeJA signaling pathways in this genotype (Figure 7c,d). Notably, transcript levels of both marker genes were higher in the wild genotype than in the domesticated cultivar. Collectively, these results indicate that *MamRGA2* and *MacRGA2* are not positively induced by SA and display differential regulation in response to MeJA, characterized by strong induction of *MamRGA2* in the wild genotype, and absence of induction of *MacRGA2*, accompanied by a slight decrease in expression in the cv. Grand Nain.

### 3.7. Comparison of MamRGA2 and MacRGA2 Promoter Regions

**Figure 8:**
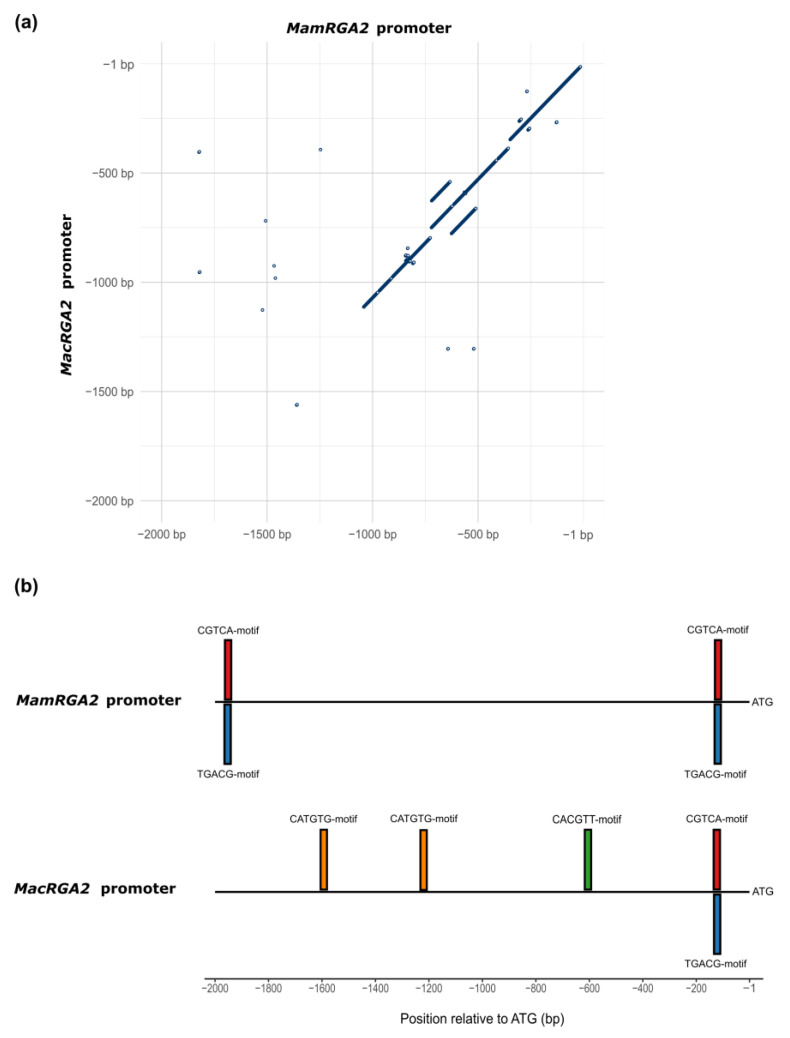
Comparative analysis of the *MamRGA2* and *MacRGA2* promoter regions. (**a**) Dot plot analysis of a 2 kb fragment corresponding to the *MamRGA2* and *MacRGA2* promoters. The main diagonal indicates highly conserved sequence regions. (**b**) Distribution of methyl jasmonate (MeJA)-responsive *cis*-regulatory elements within the *MamRGA2* and *MacRGA2* promoters.

Based on the expression results, the promoter regions of *MamRGA2* from the wild genotype *M. acuminata* ssp. *malaccensis* and *MacRGA2* from the domesticated Cavendish cultivar Baxijiao [25] were examined and compared. A 2 kb sequence upstream of the translation start codon was analyzed for each gene. For *MacRGA2* in cv. Baxijiao, the sequence BXH1_00016606-R1 was selected, as it showed the highest percentage of identity to *MamRGA2*. Dot plot analysis (Figure 8a) revealed strong structural conservation in the proximal promoter region (−1000 to −1 bp relative to the ATG), evidenced by a continuous diagonal representing aligned homologous segments. This region showed 88.11% sequence identity between genotypes. In contrast, the distal region (−2000 to −1000 bp) exhibited pronounced divergence, characterized by sparse alignment signals and a sequence identity of 24.62%.

Because *MamRGA2* was inducible by MeJA in the wild genotype but not its ortholog, *MacRGA2*, in the domesticated Cavendish cultivar, the 2 kb promoter regions of both genotypes were further analyzed using the PlantCARE database to identify potential jasmonate-responsive *cis*-elements. In the wild genotype, two copies each of the TGACG and CGTCA motifs, both associated with MeJA signaling, were detected. In contrast, the domesticated genotype contained only a single copy of each of these motifs, along with two CATGTG motifs and one CACGTT motif, also linked to jasmonate responsiveness which were absent from the wild genotype (Figure 8b).

The TGACG and CGTCA motifs correspond to binding sites for bZIP transcription factors, particularly of the TGA type, whereas CATGTG and CACGTT are recognized by bHLH transcription factors of the MYC type.

### 3.8. Three-Dimensional Structure of the Predicted MamRGA2 Resistosome

**Figure 9:**
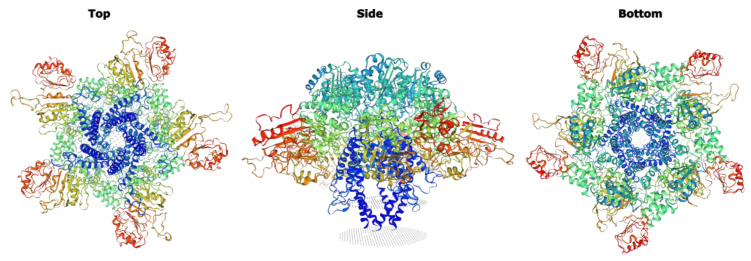
Structural prediction of the MamRGA2 resistosome. Individual domains are color-coded as follows: blue, N-terminal coiled-coil (CC) domain; green, central nucleotide-binding site (NBS) domain; and red, C-terminal leucine-rich repeat (LRR) domain. Three views of the three-dimensional model are shown to illustrate the oligomeric organization of the complex. The structure was generated by homology modeling using the SWISS-MODEL server, with the activated resistosome of the *Arabidopsis thaliana* resistance protein ZAR1 (PDB: 6J5T) as the structural template.

A predictive structural model of the MamRGA2 resistosome was generated by homology modeling using the activated pentameric ZAR1 complex from *A. thaliana* (PDB: 6j5t.L) as a template. The resulting model exhibits a symmetric assembly of five subunits, in which the CC domains are oriented toward the center of the complex, while the LRR domains extend outward (Figure 9). The predicted resistosome displays a well-defined central cavity and an overall architecture closely resembling that of the ZAR1 resistosome, with a domain arrangement consistent with the pentameric symmetry characteristic of activated CNL resistosomes.

### 3.9. Hypothetical Model of Jasmonate-Mediated Induction of MamRGA2 Expression in Resistance to Foc TR4

**Figure 10:**
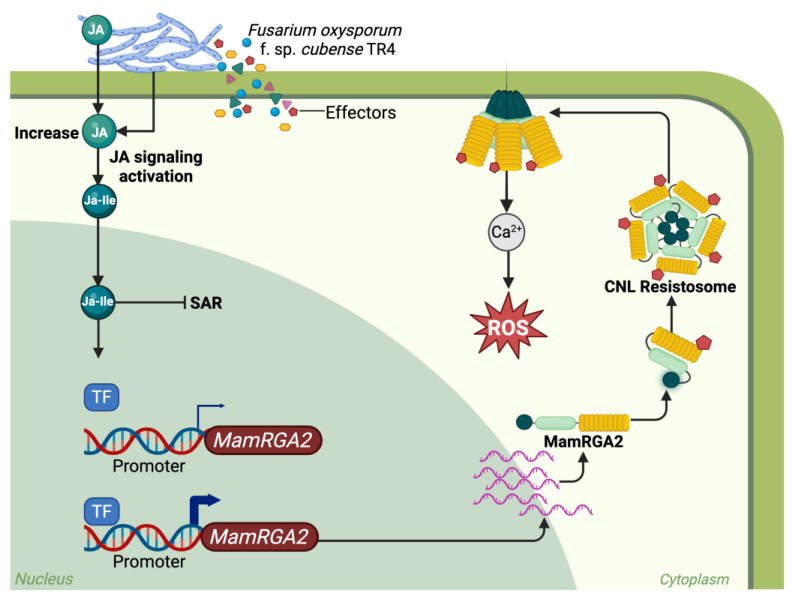
Hypothetical model of jasmonate (JA)-mediated induction of *MamRGA2* expression in resistance to Foc TR4 in *Musa acuminata* ssp. *malaccensis*. In this model, Foc TR4 may secrete jasmonates (JA; green spheres) or induce JA biosynthesis in the host plant, or both processes simultaneously. JA accumulation could antagonize the salicylic acid (SA)-mediated systemic acquired resistance (SAR) pathway, contributing to its attenuation. However, in the resistant genotype *M. acuminata* ssp. *malaccensis*, accumulation of the bioactive conjugate JA-Ile would activate the jasmonate signaling cascade, promoting transcriptional upregulation of *MamRGA2* and activation of its corresponding CNL resistance protein. This signaling cascade may lead to resistosome assembly and the activation of effective immune responses against Foc TR4, including the production of reactive oxygen species (ROS). A thin arrow preceding the upregulation of *MamRGA2* (thick arrow) indicates its basal expression in both leaf and root tissues of *M. acuminata* ssp. *malaccensis*, as previously reported by Peraza-Echeverria et al. [51,52]. Abbreviations: JA, jasmonic acid; TF, transcription factor; ROS, reactive oxygen species; CNL, CC-NBS-LRR resistance protein.

Based on the structural and expression data obtained for *MamRGA2* in this study, together with previous evidence linking jasmonate signaling to resistance against Foc TR4 [48,49], we propose a hypothetical model describing the role of jasmonic acid (JA) in the regulation of *MamRGA2* and its potential contribution to defense against this pathogen (Figure 10). In this model, JA may originate from multiple sources, including possible production by Foc TR4 (see Section 4), induction of JA biosynthesis in the host plant following infection, or a combination of both. Elevated JA levels could negatively modulate salicylic acid (SA)-mediated systemic acquired resistance (SAR) due to the well-known antagonistic crosstalk between these signaling pathways [50]. However, in the resistant wild genotype *M. acuminata* ssp. *malaccensis*, the presence of JA-responsive *cis*-regulatory elements in the *MamRGA2* promoter may enable rapid and robust transcriptional activation of this resistance gene upon infection, which may be associated with enhanced defense responses. In contrast, the susceptible Cavendish cultivars possess a distinct configuration of JA-responsive *cis*-elements in the *MacRGA2* promoter, which may result in altered transcriptional responsiveness and could represent one factor associated with their susceptibility to Foc TR4. This hypothetical model provides a conceptual framework for future studies aimed at elucidating the perception, signaling, and transcriptional regulation mechanisms underlying resistance to Foc TR4.

## 4. Discussion

This study provides a genome-wide characterization of the NBS gene family in the wild banana *M*. *acuminata* ssp. *malaccensis*, identifying 118 genes distributed across nine subfamilies. This number exceeds previous reports for version 4 of this genome, which identified 97 [27] and 116 genes [28], respectively. These differences likely reflect methodological variations among studies and suggest an improved annotation and a more comprehensive characterization of the NBS gene family in banana. In addition, the present analysis enabled the identification of three previously undescribed NBS genes in banana. Among the newly identified genes, one RPW8-NBS-LRR (RNL) member is particularly noteworthy, as this subfamily is sparsely represented in plants. In *A. thaliana*, only two RNL genes (*ADR1* and *NRG1*) have been characterized; these function as helper receptors that amplify immune signaling downstream of the TIR-NLR complex RPS4/RRS1 against pathogens such as *Erysiphe* spp. and *Hyaloperonospora arabidopsidis* [53,54]. Similarly, only a few RNL genes have been reported in *Oryza sativa* and *Zea mays*, with functions that remain largely unresolved [55,56]. The presence of an RNL in banana suggests that this signaling module may be more widespread in monocots than previously appreciated and warrants functional investigation. The remaining two novel NBS genes encode proteins containing integrated domains (IDs) corresponding to an ABC transporter and an NFYA transcription factor. The predicted subcellular localization of these proteins is consistent with the known functions of their domains. In *A. thaliana*, ABC transporters such as PEN3 and PIS1 localize to the plasma membrane and contribute to defense by secreting antimicrobial metabolites such as camalexin [57,58]. In contrast, NFYA transcription factors primarily act in the nucleus, regulating genes involved in biotic and abiotic stress responses through binding to CCAAT motifs in promoter regions [59,60]. In *Z. mays*, *ZmNFYA01* is induced upon infection by *Fusarium graminearum* and enhances resistance by modulating genes such as *ZmPR*, thereby reducing disease severity [61]. From a comparative perspective, these findings expand the known diversity of NBS proteins with integrated domains. NBS proteins carrying ABC-type IDs have only been described in *Populus trichocarpa* and *Z. mays* [62], whereas NFYA-type IDs have been reported only in certain dicots, including *Brassica napus*, *Brassica rapa*, and *Linum usitatissimum* [19]. Functionally, IDs can act as molecular decoys that mimic host effector targets, thereby intercepting pathogen virulence strategies [63]. This mechanism has been demonstrated in *A. thaliana*, where RRS1-R detects the bacterial effector PopP2 via a WRKY-type ID [64,65]. The structural plasticity of IDs also supports emerging biotechnological applications, such as engineering nanobodies into NLRs to broaden effector recognition spectra [66,67].

The clear separation between TIR and non-TIR NBS proteins in comparison with *A. thaliana* supports functional divergence and the loss of the TIR pathway in monocots [68]. Notably, phylogenetic analysis including experimentally validated *R* genes showed that eight of twelve clades contain *R* genes, suggesting conserved immune functions across species. In particular, *MamRGA2* clustered in clade XII together with tomato *I2* and close to clade XI containing melon *Fom-2*, both conferring resistance to *F. oxysporum* f.sp. *lycopercisi* and *F. oxysporum* f.sp. *melonis*, respectively. This pattern supports functional conservation among NLRs active against closely related pathogens [16,69]. Subcellular localization predictions indicated that most NBS proteins localize to the nucleus, suggesting roles in transcriptional regulation of defense genes. This observation is consistent with CNLs such as rice PID3, which interacts with the transcription factor RAI1 in the nucleus to activate defense responses [70], and with evidence that nuclear accumulation of certain CNLs is essential for immunity [71]. The plasma membrane was the second most frequent compartment, in agreement with CNLs such as RPS5 and RPM1 of *A. thaliana*, which detect pathogen-induced modifications in host proteins [72,73]. A substantial fraction of NBS proteins was also predicted in the cytoplasm, where early signaling pathways such as Ca^2+^ influx and MAPK cascades are activated [74]. MamRGA2 shares this localization with cytoplasmic CNLs such as RB and Gpa2 from potato [75,76]. Additionally, two NBS proteins exhibited multi-compartment localization (nucleus/plasma membrane and nucleus/cytoplasm/apoplast), a feature associated with functional flexibility observed in wheat PM3b and barley MLA10 [77,78]. Collectively, this diversity likely reflects adaptation to the various subcellular sites targeted by pathogen effectors.

The genomic distribution of the 118 NBS genes across the *M. acuminata* ssp. *malaccensis* genome suggests that their expansion resulted from localized evolutionary processes. The strong clustering of genes in specific chromosomal regions indicates that tandem duplication has been a major mechanism driving NBS gene amplification. Such expansion patterns are typical of plant NLR genes and are associated with recurrent cycles of duplication, diversification, and gene loss driven by host–pathogen coevolution [39,79,80]. In contrast, *MamRGA2*, which confers resistance to Foc TR4 [29,51,81], occurs as a singleton located 1.6 Mb from the nearest NBS gene. This organization resembles that of *Fom-2* in *Cucumis melo* [82], but contrasts with tomato *I2*, which resides in a cluster of seven NBS genes [83]. Such organizational differences are consistent with convergent evolutionary solutions for resistance to *F. oxysporum* [52].

The genomic neighborhood of *MamRGA2* is enriched in genes putatively associated with signaling and defense-related processes, suggesting the presence of a specialized immune-associated locus. Notably, Macma4_03_g09410, encodes a calmodulin-2 homolog, a key Ca^2+^ sensor involved in signal transduction during biotic stress [84]. This is particularly relevant given that NLR resistosomes can function as Ca^2+^-permeable channels that trigger ion fluxes upon pathogen recognition [85,86,87]. Another neighboring gene, Macma4_03_g09310, encodes a putative auxin transporter similar to WAT1 in *A. thaliana*, whose inactivation reduces susceptibility to vascular pathogens such as *Clavibacter michiganensis* [88], highlighting links between hormone homeostasis and disease resistance. The presence of Macma4_03_g09480, homologous to importin-type proteins, further suggests mechanisms for nuclear import of defense regulators [89], reinforcing coordination between cytoplasmic signaling and nuclear transcriptional reprogramming. The region also contains two genes (Macma4_03_g09180 and g09240) homologous to E3 ubiquitin ligases, which regulate NLR stability and activation [65,90], as well as Macma4_03_g09230, a putative methyltransferase implicated in epigenetic regulation of defense loci [91]. Additionally, Macma4_03_g09600, Macma4_03_g09530, and Macma4_03_g09550 encode transcription factors related to NAC, EIN3/EIL, and WUSCHEL families, respectively, all involved in hormone signaling and biotic stress responses [92,93,94]. Collectively, the coexistence of calcium-mediated, hormonal, epigenetic, proteostatic, and transcriptional regulators suggests that *MamRGA2* resides within a specialized genomic microenvironment associated with immune responses. Moreover, microsynteny analysis revealed not only high structural conservation between *M. acuminata* ssp. *malaccensis* and *M. acuminata* cv. Baxijiao, but also loss of four genes in cv. Baxijiao, including the importin homolog (Macma4_03_g09480). Such gene loss may reflect domestication processes [95] and could have functional consequences for immune competence in this cultivar.

Expression analyses showed that *MamRGA2* and *MacRGA2* respond differentially to MeJA. *MamRGA2* is strongly induced by MeJA in the wild genotype *M. acuminata* ssp. *malaccensis* but this is not the case for *MacRGA2* in *M. acuminata* cv. Grand Nain. This difference correlates with divergence in promoter architecture and supports the proposed model (Figure 10), in which JA-responsive *cis*-elements could facilitate the activation of *MamRGA2* in the resistant genotype, whereas altered promoter configuration of its ortholog *MacRGA2* in cv. Grand Nain may result in delayed or insufficient defense responses against Foc TR4. Reduced gene inducibility in domesticated cultivars may reflect selection for agronomic traits at the expense of immune responsiveness, a pattern documented in multiple crop species [96,97,98,99]. Pathogen-mediated manipulation of JA signaling may further influence this interaction. Some *F. oxysporum* formae speciales have been reported to produce JA or JA-like compounds [100,101], raising the possibility that similar mechanisms could also occur in Foc TR4, potentially disrupting hormonal cross-talk with salicylic acid to suppress systemic acquired resistance. Indeed, many fungal pathogens exploit hormone signaling pathways to promote susceptibility [102,103]. Alternatively, Foc TR4 may stimulate host JA signaling without producing JA itself. For example, Foc R1 induces JA-related genes in cv. Grand Nain and confers protection against subsequent Foc TR4 infection [49]. Repeated MeJA treatments on the leaves of the *M. acuminata* cv. Williams (Cavendish cultivar) similarly induce systemic resistance against Foc TR4 [48]. These findings reinforce the central role of JA signaling in banana immunity and support the biological relevance of *MamRGA2* inducibility in a Foc TR4 resistant genotype. Consistent with this view, the nopaline synthase (nos) promoter from *Agrobacterium tumefaciens*, used to drive *MamRGA2* expression in transgenic Grand Nain plants resistant to Foc TR4 [29,81], is MeJA-inducible [104]. Thus, JA-responsive transcriptional activation of *MamRGA2* may contribute to the activation of defense responses when its expression is appropriately regulated. The native promoter of *MamRGA2* in the wild genotype may therefore represent a candidate regulatory region associated with JA responsiveness during pathogen challenge. Candidate JA-responsive cis-regulatory elements identified in the promoter regions of *MamRGA2* and *MacRGA2* include motifs recognized by transcription factors that can function as either positive or negative regulators of jasmonate-responsive gene expression [105]. These findings highlight promoter regulation as a promising area for future research on this disease resistance gene. In particular, CRISPR-based promoter editing approaches [106,107] could be employed to assess whether targeted editing of JA-responsive cis-regulatory elements in the *MacRGA2* promoter influences gene inducibility and defense responses to Foc TR4 in Cavendish cultivars.

## 5. Conclusions

This study provides a comprehensive update on the diversity and genomic organization of NBS genes in banana. For the first time, two NBS genes harboring integrated domains corresponding to an ABC transporter and an NF-YA transcription factor were identified in this species. We also identified an RNL-type gene, previously unreported in banana, broadening the repertoire of NBS subfamilies present in the *Musa* genome. Chromosome 3 stood out due to its high concentration of NBS genes and the presence of the resistance gene *MamRGA2*, underscoring its relevance in defense against Foc TR4 and possibly other phytopathogens. Notably, this gene was inducible by MeJA in the wild genotype *M. acuminata* ssp. *malaccensis* but not in the domesticated cv. Grand Nain, which may be associated with differences in promoter architecture. These results open new avenues for investigating the functional roles of NBS genes containing integrated domains and the regulatory mechanisms underlying MeJA-inducible *MamRGA2* expression. Such efforts may provide new insights into disease resistance mechanisms in banana and support novel breeding strategies against Foc TR4 and other phytopathogens.

## Figures and Tables

**Table 1:** Classification and number of NBS-encoding genes identified in the genome of *Musa* acuminata ssp. *malaccensis*.

Classification	Letter Code	Number	%
CC-NBS-LRR	CNL	79	66.95
NBS-LRR	NL	17	14.41
NBS	N	9	7.63
CC-NBS	CN	7	5.93
CC-NBS-LRR-NBS-LRR	CNLNL	2	1.69
NBS-LRR-CC-NBS-LRR	NLCNL	1	0.85
RPW8-NBS-LRR	RNL	1	0.85
ABCtrans-CC-NBS-LRR	ID-CNL	1	0.85
CBFB/NFYA-CC-NBS-LRR	ID-CNL	1	0.85
	**Total NBS genes**	**118**	**100**
	Total predicted genes	36,769	
	% NBS genes	0.32	
	Genome size (Mb)	523	

## Data Availability

The identification and analysis of NBS genes were conducted using publicly available genomic resources. The genome sequences of *M. acuminata* ssp. *malaccensis* (v4) and *M. acuminata* cv. Baxijiao were retrieved from the Banana Genome Hub database (https://banana-genome-hub.southgreen.fr/). All data generated or analyzed during this study are included in this article and its Supplementary Materials.

## Supplementary Materials

The following supporting information can be downloaded at: https://www.mdpi.com/article/10.3390/genes17060700/s1, Figure S1. Conserved motifs of 118 NBS proteins from Musa acuminata ssp. malaccensis; Table S1. NBS genes identified in Musa acuminata ssp. malaccensis; Table S2. List of 70 cloned R genes from monocot and dicot plant species used for phylogenetic analysis; Table S3: Chromosomal distribution and physical positions of NBS genes in Musa acuminata ssp. malaccensis; Table S4. List of 50 genes flanking *MamRGA2* in *Musa acuminata* ssp. *Malaccensis* [108,109,110,111,112,113,114,115,116,117,118,119,120,121,122,123,124,125,126,127,128,129,130,131,132,133,134,135,136]; Table S5. Primers used for RT-qPCR analysis.
