# Supplementary material for: Genome-Wide Analysis of the Banana NBS Gene Family and Expression Profiling of the Fusarium Wilt Resistance Gene MamRGA2 in Response to Defense-Related Phytohormones
Source: Genes (Basel). 2026 Jun 16;17(6):700. doi: 10.3390/genes17060700 (PMC13300587; doi:10.3390/genes17060700)
Supplement: Supplementary file 1 [file genes-17-00700-s001.zip › Supplementary figure S1 Roblero-Aguilar et al..pdf]

Supplementary Materials

Genome-wide analysis of the banana NBS gene family and expression profiling of the Fusarium wilt resistance gene *MamRGA2* in response to defense-related phytohormones

Ana Nury Roblero-Aguilar[1], Gabriel Lizama-Uc[2], Carlos Alberto Puch-Hau[3], Virginia Aurora Herrera-Valencia[1], Sergio García-Laynes[1], Jorge Antonio Tzec-Interián[1], Marta G. Lizama-Gasca[1], Ileana Cecilia Borges-Argaez[1], and Santy Peraza-Echeverria[1]*

*Correspondence: santype@cicy.mx; Tel.: +52-9999-428-330

Supplementary figures

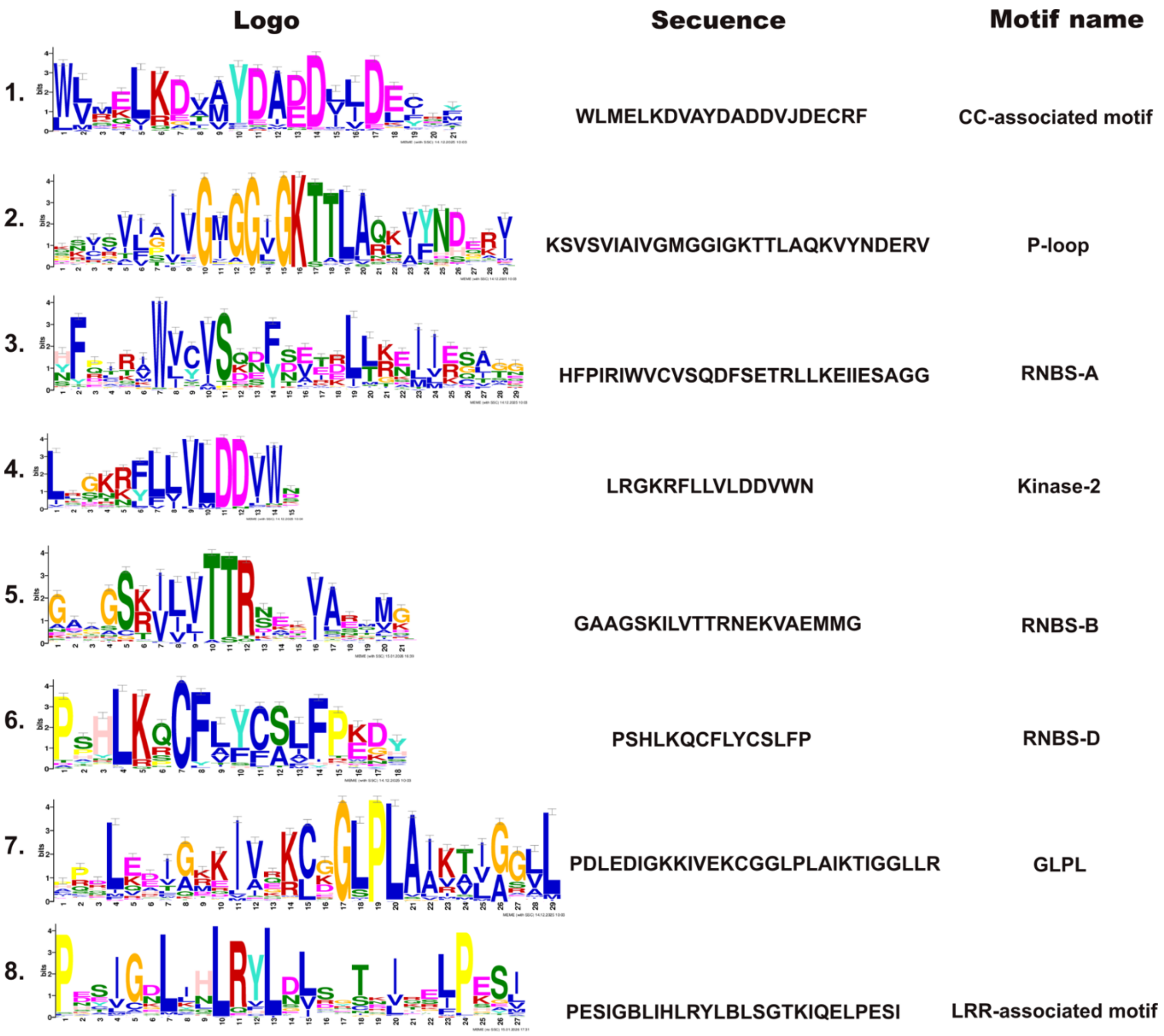

Supplementary Figure S1. Conserved motif analysis of 118 NBS proteins from *Musa acuminata* ssp. *malaccensis*. Sequence logos of the eight identified conserved motifs are displayed, showing their consensus sequences and motif designations. Motif identification was conducted using MEME
